# Supplementary material for: Impact of an acceptance facilitating intervention on psychotherapists’ acceptance of blended therapy
Source: PLoS One. 2020 Aug 12;15(8):e0236995. doi: 10.1371/journal.pone.0236995 (PMC7423074; doi:10.1371/journal.pone.0236995)
Supplement: S1 File — (DOCX) [file pone.0236995.s001.docx]

Video script – An acceptance facilitating intervention for blended therapy

**Szene 1: *Folie*** *wird eingeblendet mit dem Titel „blended therapy – Eine Studie der Universität Ulm in*

*Kooperation mit der Landespsychotherapeutenkammer Baden-Württemberg“.*

**Szene 2:** *Spielt im* ***Therapiezimmer der Psychotherapeutischen Hochschulambulanz (PHSA) der Universität***

***Ulm****. Anwesend sind eine Therapeutin und eine Patientin.*

*Therapeutin:* Heute haben wir über Ihre Probleme und Ihren Wunsch gesprochen, in Leistungssituationen selbstsicher zu sein und zum Beispiel einen Vortrag halten zu können. Wir haben uns gemeinsam in eine solche Situation hineinversetzt und sie durchgespielt.

*Patientin:* Ja. Und ich habe auch heute festgestellt, wie schwer mir so ein spontaner Vortrag fällt. Ich möchte wieder selbstsicherer sein und auf Menschen zugehen können. Und ich möchte auch keine Angst mehr haben, wenn ich vor anderen Menschen sprechen muss.

*Therapeutin:* Ich denke es könnte Ihnen helfen, wenn Sie lernen die Aufmerksamkeit mehr auf Ihre Umwelt zu richten – und weniger auf Sie selbst. Das ist nicht ganz so einfach, aber wir werden das gemeinsam üben. Im ersten Schritt möchte ich Sie bitten sich bis zur nächsten Sitzung mit dem Thema vertraut zu machen. Auf unserer Online-Plattform habe ich Ihnen dafür wieder Informationen und erste Übungen freigeschaltet. Nächste Woche sprechen wir dann darüber, wie es Ihnen ergangen ist. Dann sehen wir uns wieder nächste Woche Mittwoch um 15 Uhr?

*Patientin:* Ja, ok.

*Therapeutin:* Alles klar, dann bis nächste Woche, Frau Herzog. Alles Gute bis dahin.

*Beide stehen auf und schütteln sich die Hand zur Verabschiedung.*

**Szene 3:** *Experte im* ***Büro****. Eingeblendet wird „Harald Baumeister - Psychotherapeut und Professor für*

*Klinische Psychologie und Psychotherapie an der Universität Ulm“.*

*Experte:* Blended therapy – verzahnte Therapie. Darunter verstehen wir die Integration onlinebasierter Elemente in unsere psychotherapeutische Arbeit vor Ort. Die Einsatz- und Nutzungsmöglichkeiten sind vielfältig. Wir können unseren Patienten z. B. Online-Übungen, Psychoedukation oder Entspannungsverfahren zur Verfügung stellen und diese in der nächsten Therapiesitzung mit unseren Patienten besprechen.

*Ein Ausschnitt der Intervention GET.ON Panik wird eingeblendet.*

*Experte:* Es ist auch möglich, vollständige Interventionen für Problembereiche zu nutzen, für die vor Ort nicht ausreichend Zeit bleibt. Manchmal können Patienten aus verschiedenen Gründen, weil sie zum Beispiel geh-eingeschränkt aufgrund eines Unfalls sind, nicht zu uns vor Ort in die Psychotherapie kommen. In solchen Fällen kann es hilfreich sein, per gesicherter E-Mail, Chat oder Videotelefonie mit unseren Patienten in Kontakt treten zu können.

**Szene 4:** *Patientin sitzt* ***zu Hause*** *am PC und bearbeitet eine Online-Übung. Dann dreht sie sich zur*

*Kamera.*

*Patientin:* Was ich besonders schätze ist die Flexibilität. Ich kann das Programm von zu Hause aus nutzen, wann immer ich Zeit dafür habe. Außerdem gibt mir meine Therapeutin die Möglichkeit bestimmte Themenbereiche eigenständig zu vertiefen. So fühle ich mich auch außerhalb der Therapiesitzungen gut betreut.

**Szene 5:** *Experte im* ***Büro*** *(währenddessen werden Stichpunkte eingeblendet).*

*Experte:* Ein weiterer Vorteil von blended therapy ist, dass die Selbstwirksamkeit und die Eigenverantwortung unserer Patienten gestärkt werden. Denn die Patienten nehmen bei der Bearbeitung der Online-Interventionen eine aktive Rolle ein. Durch die Nutzung von internetbasierten Interventionselementen bleibt uns mehr Zeit für die eigentliche psychotherapeutische Prozessarbeit. Patienten können sich zum Beispiel zu Hause einfach zu vermittelndes, aber für die Therapie wichtiges Wissen aneignen, welches wir ansonsten zeitaufwendig in unserer Therapie hätten vermitteln müssen. Als abschließendes Beispiel für die Nutzung von blended therapy können internetbasierte Interventionen auch zur Überbrückung der Wartezeit eingesetzt werden. Derart tragen wir zur Verbesserung der Versorgungssituation bei und gleichzeitig können wir unsere Patienten so gezielt auf unsere Therapie vor Ort vorbereiten.

**Szene 6:** *Patientin, sitzt* ***zu Hause*** *am PC.*

*Patientin:* Falls technische Schwierigkeiten auftreten, wird ein Support von den Betreibern der Plattform angeboten. Die Bedienung ist wirklich einfach zu handhaben: Man kann diese onlinebasierten Interventionen auch ohne Vorerfahrung mit Computern und dem Internet verwenden. Außerdem finde ich sehr gut, dass meine Daten verschlüsselt gespeichert werden und somit vor unbefugten Zugriffen geschützt sind.

**Szene 7:** *Vor und in der* ***PHSA:*** *Patientin steigt vom Fahrrad, stellt es ab, betritt das Gebäude und läuft die Treppen zur Ambulanz hoch.*

**Szene 8:** *Patientin sitzt im* ***Wartebereich der PHSA*** *und wird von der Psychotherapeutin abgeholt.*

*Therapeutin:* Hallo Frau Herzog! Kommen Sie bitte mit.

*Patientin:* Hallo.

**Szene 9:** *Spielt im* ***Therapiezimmer der PHSA.*** *Anwesend sind die Therapeutin und die Patientin.*

*Therapeutin:* Dann bitte setzen Sie sich. Erzählen Sie mal: Wie ist es Ihnen denn mit Ihrer internetbasierten Übung vergangene Woche ergangen? *Patientin:* Das war wirklich interessant. Ich habe versucht mich im Gespräch mit einer Freundin mehr auf die Außensituation zu konzentrieren und weniger auf meine Gedanken und Ängste.

*Therapeutin*: Toll, dass Sie sich darauf einlassen konnten und erfolgreich waren! Das freut mich sehr für Sie! Wie war es denn für Sie, sich im Gespräch mehr auf die Umwelt zu konzentrieren?

*Patientin:* Ja also anfangs war das gar nicht so leicht. Ich habe noch viel darüber nachgedacht, wie ich im Gespräch wirke. Das war in der Internetübung auch gut beschrieben. Da habe ich mich gleich wiedererkannt. Aber ich habe das Treffen mit meiner Freundin so auch ganz anders wahrgenommen. Ich glaube ich war aufmerksamer und konnte ihr besser zuhören...

*Patientin redet weiter, das Bild wird ausgeblendet.*

**Szene 10: *Folie*** *wird eingeblendet mit dem Titel „blended therapy – Eine Studie der Universität Ulm in*

*Kooperation mit der Landespsychotherapeutenkammer Baden-Württemberg“.*
