# Supplementary material for: Impact of an acceptance facilitating intervention on psychotherapists’ acceptance of blended therapy
Source: PLoS One. 2020 Aug 12;15(8):e0236995. doi: 10.1371/journal.pone.0236995 (PMC7423074; doi:10.1371/journal.pone.0236995)
Supplement: Attachment — Submitted filename: Baumeister_Blended Therapy AFI_P2P reply.docx [file pone.0236995.s003.docx]

# Point-to-point reply to reviewers´ comments on our manuscript:

“Impact of an acceptance facilitating intervention on psychotherapists’ acceptance of blended psychotherapy”

Substantial changes are green marked in the revised manuscript.

# Editorial

1. Please ensure that your manuscript meets PLOS ONE's style requirements, including those for file naming. The PLOS ONE style templates can be found at

http://www.journals.plos.org/plosone/s/file?id=wjVg/PLOSOne_formatting_sample_main_body.pdf and http://www.journals.plos.org/plosone/s/file?id=ba62/PLOSOne_formatting_sample_title_authors_affiliations.pdf

**Answer**: We have formatted the manuscript according to the provided style templates of PLOS ONE.

2. We note that you have indicated that data from this study are available upon request. PLOS only allows data to be available upon request if there are legal or ethical restrictions on sharing data publicly. For more information on unacceptable data access restrictions, please see http://journals.plos.org/plosone/s/data-availability#loc-unacceptable-data-access-restrictions.

In your revised cover letter, please address the following prompts:

a) If there are ethical or legal restrictions on sharing a de-identified data set, please explain them in detail (e.g., data contain potentially sensitive information, data are owned by a third-party organization, etc.) and who has imposed them (e.g., an ethics committee). Please also provide contact information for a data access committee, ethics committee, or other institutional body to which data requests may be sent.

b) If there are no restrictions, please upload the minimal anonymized data set necessary to replicate your study findings as either Supporting Information files or to a stable, public repository and provide us with the relevant URLs, DOIs, or accession numbers. For a list of acceptable repositories, please see http://journals.plos.org/plosone/s/data-availability#loc-recommended-repositories.

 We will update your Data Availability statement on your behalf to reflect the information you provide.

**Answer**: The anonymized dataset necessary to replicate our study findings is provided as supporting information file, after clarifying the data security issues with our legal department.

3. Please include a caption for figure 3.

**Answer:** We included a list of all figures following the references in the manuscript. All figures are also listed within the manuscript. A caption was added to figures 2 and 3.

# **Reviewer #1:**

1. This is an informative paper that addresses an important aspect of psychotherapeutical practice. The authors investigate how an acceptance-facilitating intervention affects psychotherapists' acceptance of blended therapy. Since internet- and mobile-based interventions are becoming an increasingly important element of psychotherapy, this research is highly relevant.

**Answer**: Thank you!

2. The methodology is sound, what is missing is a brief discussion of the current state of research on the topic. The authors claim that their study is the first on acceptance-facilitating interventions regarding psychotherapists' acceptance of blended therapy. However, there are several studies on the attitudes vis-a-vis blended therapy and the acceptance thereof among psychotherapists (e.g. Schuster et al. 2018: <https://www.jmir.org/2018/12/e11007/>; Mayer et al. 2019: <https://mental.jmir.org/2019/11/e14018/>). Although the study presented by the authors is methodologically more sophisticated than many of the existing studies, which are mainly survey-based, the authors should mention the existing research on the topic. This helps the reader to better understand the relevance of the paper and it also shows the innovativeness of the study. A few short sentences and two or three sources will suffice.
   **Answer**: We incorporated the mentioned studies as well as further studies into the manuscript.
3. As a minor point, I would suggest a language check by a native speaker. All in all, the language and style are appropriate, but at some points, the wording is odd or wrong (e.g. "normal distributed" instead of "normally distributed")

**Answer**: To ensure high quality in language and style the manuscript was proofread by a native speaker.

# Reviewer #2:

1. This is an interesting paper about the integration of technology with the traditional face-to-face way to work in psychotherapy. It could be an initial point to understand this phenomenon. The reduced psychotherapist sample and the absence of a pre-post analysis are the main problems of the research. Additionally there are different parts of the research needed of some explanation to improve the understanding of the paper.

**Answer**: We would like to thank the reviewer for his encouraging statement and the work put into this review. Based on the reviewer’s remarks we were able to substantially improve the manuscript. Our revisions to the itemized points are outlined below.

2. The abstract inform that one of the aims of the study was to identify potential effect moderators. The analysis of moderators is more complex than a regression analysis. The use of this term could be generate confusion.

**Answer**: As outlined by the reviewer we used regression model to identify moderators. We included the main effects (e.g. group and sex) as well as the interaction effects (e.g. group*sex) in the models. Interpretation-wise the main effects indicate the influence on the depending variable (=acceptance), while the interaction effect indicates the influence above the main effects. In a hypothetical example results are beta=.5 for the group main effect, beta=.2 for the sex [0=male, 1=female) main effect and beta=.3 for the interaction effect. This would mean 1. that the group has an positive effect of 0.5 on the acceptance, 2. that females in general show an higher acceptance (difference to males: 0.2) and 3. that the intervention effect is higher in females (combined effect would be 0.5 + 0.3=0.8). We think this procedure is well suited to identify potential moderators (e.g. sex in the hypothetical example). However, we do agree that the terms regression analysis and exploratory analysis should be used in a clearer way. Accordingly, we revised the method section and results section to avoid any confusions.

3. They informed the use of an online questionnaire. It is no clear if is an ad hoc questionnaire or a well know questionnaire with an adequate validity and reliability.

**Answer**: We thank the review for pointing out this potential source of confusion for the reader. As introduced in the present manuscript the questionnaire is adapted from the questionnaire proposed in the UTAUT model by Venkatesh and colleagues. The questionnaire has been successfully used in several previous studies. In a different project we have investigated the psychometric quality of the instrument. The model fit of the instrument is excellent: RMSEA=0.047, SRMR=0.033, CFI=0.978 and TLI=0.970. However, the manuscript is still in preparation and thus, we currently cannot provide a reference.

To avoid any confusions we changed the section to:

“Both groups received a reliable online questionnaire assessing acceptance, effort expectancy, performance expectancy, facilitating conditions, social influence and internet anxiety”

4. In the introduction paragraph (line 55), they uses the acronym RR with no reference to his meaning.

**Answer**: RR is the commonly used abbreviation for relative risk. To make this clearer we put the statistical figures directly behind the mentioning of relative risk. The revised passage reads as follows:

“Moreover, the pooled relative risk (RR = 2.22, 95%-CI: 2.12-2.33) of mortality among persons with mental disorders is increased by over 120 % compared to those without mental disorders [6].”

5. There is a contradiction between the content of IG video. It is a video showing case (line 108) or a presenting information about blended psychotherapy (line 121)? How is produce the video: with real psychotherapist, actors, etc.? The video is not accessible to the URL provided. Nevertheless, the IG video has a positive orientation and the placebo video a clear negative orientation. Could these aspects affect to the results?

**Answer**: Thanks for highlighting this issue. In the submitted version the quoted sentences are redundant to the in-depth description of the videos in the section “experimental conditions”. We changed the procedure descriptions and referenced to the “experimental conditions” section.
“The IG received an AFI video and the CG received a placebo video. Detailed information on the intervention and control video are provided the section “experimental conditions” below.”

All information on how the video was shot (e.g. role play by actors) are presented in the experimental conditions section. The online links have been updated.

6. The primary outcome is about “blended psychotherapy”. Is it a term well known by the German psychotherapist?

**Answer**: The term “blended psychotherapy” has been introduced and described to the participants (= German psychotherapists) in detail prior to the outcome assessment.

7. There are a difference in acceptance baseline between CG and IG groups (line 226-229). A t-test show a significant difference t= 2.131, 231 d.f. p>.025. If there are a difference between groups before the experimental manipulation, the results could be reconsidered.

**Answer**: The present study design is the following:

| Randomization | CG | Placebo Video | Measurement |
| --- | --- | --- | --- |
|  | IG | AFI Video | Measurement |

Based on the study design acceptance is assessed only once in both groups. The CG received a video expected to have no influence on the acceptance. The acceptance in the CG is assumed to represent the acceptance in the general population of psychotherapists. In contrast, the IG received a video expected to influence the acceptance. Since randomization was conducted before the experimental manipulation, we are assuming there are no systematic differences between CG and IG before the manipulation. Thus, the difference in acceptance measured after the experimental manipulation represents the effect of the AFI, taking the assumption of no baseline imbalance into account when interpreting our findings (as outlined in the limitation section of our discussion).

Given this design the term baseline acceptance in the cited section does not make sense and could cause confusion. To prevent any misunderstanding we removed the term baseline acceptance throughout the manuscript. We further revised the discussion section on this.

“Overall, most psychotherapists showed a moderate to high acceptance in the CG, with an average acceptance substantially above the scale mean. Given the placebo video had no effect on the acceptance, we assume that the acceptance in the CG represents the acceptance in the general psychotherapist population

8. The authors use the title “effect on acceptance” (line 235) but this term is confused. If there is not a pre-post analysis is not correct to use the term “effect”. This confusion is also present in the “effect on secondary outcomes” paragraph.

**Answer**: All participants have been randomized to either CG or IG. Thus, we assume no systematic difference between CG and IG. Thus, after the experimental manipulation (placebo vs. intervention video) differences in the acceptance or secondary outcomes can only be caused by the videos. All other casual links have been capped by randomization (that´s the standard rational of experiments using randomization). Since, the placebo video is assumed to have no influence on the acceptance towards blended therapy, we argue that the difference in the acceptance (or secondary outcomes) represents the between-group effect of the intervention compared to the CG.
We totally agree, that there is no pre-post comparison and thus, no within-group effects can be calculated. We changed all headings to between-group effect(s) to highlight, that we are referring to between-group effects and not to within group effect sizes. The lack of a pre-test is discussed in the limitation section of our manuscript (note that a pre-post test design in such an experiment with a very short intervention would have potential methodological shortcomings as well).

9. There is not specific information about the number of variables introducing in the regression analysis. It is important because the higher the number, the higher the variance explained, but there are a specific ratio between variables and cases.

**Answer**: We thank the reviewer for pointing out this missing information. We revised the methods section to provide the missing details.

“A linear regression model was used in the explorative analysis to identify moderators on the effect of AFI. The variables age, gender, therapeutic background (behavioral psychotherapy, depth psychotherapy, psychodynamic psychotherapy, other), type of license (psychological psychotherapist, child and adolescents psychotherapist, other) and their interactions with group were inserted in an initial model (all variables were effect coded or z-standardized). Group was dummy-coded (1 = IG). The initial model included a total of 20 predictors, inclusive the intercept and all interactions. In a stepwise procedure non-significant predictors were removed from the model until a final model was achieved. Ordinary Least Square (OLS) estimator was used.”

We thank all reviewers for their helpful comments!
