# Supplementary material for: Impact of an acceptance facilitating intervention on psychotherapists’ acceptance of blended therapy
Source: PLoS One. 2020 Aug 12;15(8):e0236995. doi: 10.1371/journal.pone.0236995 (PMC7423074; doi:10.1371/journal.pone.0236995)
Supplement: Attachment — Submitted filename: Baumeister_Blended Therapy AFI_P2P reply revision2.docx [file pone.0236995.s004.docx]

# Point-to-point reply to reviewers´ comments on our revised manuscript:

“Impact of an acceptance facilitating intervention on psychotherapists’ acceptance of blended psychotherapy”

Changes in the revised manuscript are highlighted.

Reviewer #1: All comments have been addressed by the authors. The paper is acceptable for publication in its present form

Note: Thanks for your time and your helpful comments!

Reviewer #3: This is an interesting article to read, pointing to the importance of acceptance for blended psychotherapy in the treatment giver rather than receiver. Authors have made an effort to improve the manuscript by carefully considering previous reviewers comments. However, there are some points that I think still needs to be addressed.
1. Measures. The questionnaire used in this article, as well as its origins from UTAUT should be more thoroughly described in the method section. Readers who are unfamiliar with this instrument should be provided with enough information to evaluate the applicability of it. For instance, it would be helpful if you added more details on the original instrument, such as total amount of items and number of factors. Please describe how items (or factors) were chosen from the UTAUT, and in what manner they were adapted from the original version (such as; if items are rephrased completely, or adapted by changing some words in the items, and why inverted items were added). If your version is currently being evaluated for psychometric properties, it would be helpful if you mention this in the manuscript. Further, it is unclear where the items on internet anxiety were derived from; the UTAUT or developed by authors?

Note: Added to the manuscript (see amendments to “Measures – paragraphs “Primary outcome” and “secondary outcome”).

Since the result section describes deviance from the scale mean, the scale mean should also be stated here (or in the result section if you are referring to the mean of the study sample).

Note: Added to the manuscript (mean and range)

2. Materials and methods. It should be more clearly stated when data was collected, this will make the result section easier to understand, that results are exploring differences after manipulation, not baseline. (You provided this clearly in your answer to reviewer 2, however it is not as clearly described in the article).

Note: Added to the methods section (p. 8 “Acceptance was assessed after participants watched the intervention (IG) or attention placebo video (CG).”)

3. Data analysis. Authors describe splitting acceptance in three categories (low/medium/high), for descriptive purposes. Could you clarify how you decided on these cut-offs? Are they based on the distribution of responses? Or theoretically derived? Is there any reason not to analyze difference in distributions on acceptance statistically? I think that a chi-square test would be informative on difference in level of acceptance. (seems to be a typo in the medium range where I suppose the range is above 9 and below 16? line 189)

Note: we added chi-square. The authors, corresponding to prior publications on this scale, have defined the cut-off values based on a theoretical basis. We added this information to the manuscript.

4. In the result section you start by describing a rather high dropout rate in the intervention group. I think the article would benefit from an analysis on difference in characteristics between those who dropped out and those who were completers. This would give a hint if there is a bias in the study sample, if those who dropped out were mostly men, mostly from a specific therapeutic background and so on.

Note: We additionally ran all analysis based on multiple imputed data. For the imputation models we followed the recommendations by van Buuren and Groothuis-Oudshoorn (2011). Imputation models included: predictors included based on theoretical considerations, correlations between variables and correlations with non-response. Given this procedure gender or therapeutic background (or any other variable) which is associated with drop-out was included in the imputation. Since, the analysis using multiple imputations yielded no difference a systematic bias/ influence is unlikely.

van Buuren S, Groothuis-Oudshoorn K. mice : Multivariate Imputation by Chained Equations in R. J Stat Softw [Internet]. 2011;45(3).

5. In Table 2 it seems as if there is a difference in distribution of gender between groups, with a higher ratio of women to men in the intervention group as compared to control group. Have you checked if this (or other demographics) is a statistically significant difference in distribution? Thoughts on implications if so? (In my version the tab-spacing made table 2 almost uninterpretable, be sure to double-check this before publication).

Note: We did not run statistical tests examining potential baseline imbalance, thereby following CONSORT statement recommendations (Moher et al. 2010). However, we agree that there might be a gender imbalance, hence, we added this potential risk of bias to the limitation section of our manuscript.

Moher et al. ConSoRT 2010 explanation and elaboration: updated guidelines for reporting parallel group randomised trials. BMJ 2010;340:c869

*p. 17: “Third, there might be a baseline imbalance between the experimental conditions regarding gender, which could have biased the present findings. As the more important it seems to replicate the present findings in further experimental studies in order to substantiate the present evidence.”*

6. The concept of blended psychotherapy and blended therapy are used interchangeably throughout the manuscript (also blend therapy, line 93). I think it would enhance the readability if authors clarify what they refer to with both concepts, or to stick with only one of them.

Note: we now use the term “blended therapy” throughout the manuscript.

7. Also as a minor point on the fluency of language; the sentence in line 75 – 79 I would consider rephrasing. Line 89-90 “it seems…” As a reader I am confused as to what “it” refers to. It is probably better to use AFIs/blended psychotherapy/ psychotherapists.

Note: Changed, thanks.

Thanks to all reviewers for their valuable time and insights!
